# Perinatal HIV Infection or Exposure Is Associated With Low *N*-Acetylaspartate and Glutamate in Basal Ganglia at Age 9 but Not 7 Years

**DOI:** 10.3389/fnhum.2018.00145

**Published:** 2018-05-07

**Authors:** Frances C. Robertson, Martha J. Holmes, Mark F. Cotton, Els Dobbels, Francesca Little, Barbara Laughton, André J. W. van der Kouwe, Ernesta M. Meintjes

**Affiliations:** ^1^Medical Imaging Research Unit, Division of Biomedical Engineering, Department of Human Biology, Faculty of Health Sciences, University of Cape Town, Cape Town, South Africa; ^2^Family Clinical Research Unit, Department of Paediatrics and Child Health, Tygerberg Children’s Hospital and Faculty of Health Sciences, Stellenbosch University, Stellenbosch, South Africa; ^3^Department of Statistical Sciences, Faculty of Sciences, University of Cape Town, Cape Town, South Africa; ^4^A. A. Martinos Centre for Biomedical Imaging, Department of Radiology, Massachusetts General Hospital, Boston, MA, United States; ^5^Department of Radiology, Harvard Medical School, Harvard University, Boston, MA, United States

**Keywords:** HIV, early ART initiation, NAA, glutamate, magnetic resonance spectroscopy, basal ganglia, HIV exposure

## Abstract

Abnormalities of the basal ganglia are frequently seen in HIV-infected (HIV+) children despite antiretroviral treatment (ART) initiation during childhood. Assessment of metabolites associated with neuronal integrity or with glial proliferation can present a sensitive description of metabolic events underlying basal ganglia structural changes. We used magnetic resonance spectroscopy to examine differences in creatine, choline, *N*-acetylaspartate (NAA), glutamate, and myo-inositol between HIV+ children and HIV-unexposed controls, as well as between HIV-exposed uninfected (HEU) children and HIV-unexposed controls at age 7 and at age 9. No differences in metabolites relative to the HIV-unexposed control group were found at age 7. However, at 9 years, both HIV+ and HEU had lower NAA and glutamate than unexposed control children. HEU children also had lower creatine and choline than control children. At age 7, lower CD4/CD8 ratio at enrollment was associated with lower choline levels. At age 9 lower CD4/CD8 at enrollment was associated with lower myo-inositol. Low NAA and glutamate at age 9, but not 7, suggest that basal ganglia neurons may be particularly affected by perinatal HIV/ART and that neuronal damage may be ongoing despite early ART and viral suppression. Reduced basal ganglia metabolite levels in HEU children suggest an effect of HIV exposure on childhood brain development that merits further investigation using neuroimaging and neurocognitive testing.

## Introduction

Although infants with perinatal HIV infection can now expect to survive childhood, they face life-long treatment with antiretrovirals (ARVs) in order to maintain a healthy immune system. Starting antiretroviral therapy (ART) before 3 months of age yields good clinical, immunological, and developmental outcomes (Violari et al., 2008; Laughton et al., 2012; Cotton et al., 2013; Crowell et al., 2015). However, it is known that ART cannot completely reverse the neurological effects of HIV (Laughton et al., 2013; van Arnhem et al., 2013; Whitehead et al., 2014) and neurodevelopmental delay and neurocognitive deficits remain (Govender et al., 2011; Donald et al., 2014; Wilmshurst et al., 2014; Musielak and Fine, 2015). In addition, neurotoxic effects of ART itself (Robertson et al., 2012) may be detrimental to brain development.

Abnormalities of the basal ganglia are frequently seen in HIV-infected (HIV+) children and adults (George et al., 2009; Hoare et al., 2014), and subcortical structures may contain the highest levels of the virus (Tornatore et al., 1994). Neuroimaging shows that HIV+ children may have shape and volume alterations in subcortical gray matter (Lewis-de los Angeles et al., 2016; Yadav et al., 2017) and calcification of the basal ganglia (Govender et al., 2011), despite ART initiation during childhood.

Magnetic resonance spectroscopy (MRS) measures brain metabolites including creatine (Cr), involved in energy metabolism, *N*-acetyl-aspartate (NAA), reflecting neuronal integrity, and glutamate (Glu), the primary excitatory neurotransmitter, as well as choline (Cho) and myo-Inositol (Ins). Ins is considered a glial marker and Cho reflects cell membrane turnover – both present in larger concentrations in glial cells than in neurons. Assessment of metabolites associated with neuronal integrity — NAA and Glu, and those associated with glial proliferation — Ins and Cho, can present a sensitive description of metabolic events underlying, and even preceding, the structural changes in basal ganglia seen in HIV+ children.

Early single voxel spectroscopy (SVS) studies in children with HIV encephalopathy (HIVE) found lower NAA/Cr ratios in the basal ganglia compared to HIV+ children without encephalopathy and controls (Pavlakis et al., 1995; Lu et al., 1996). In earlier studies where most HIV+ children did not have encephalopathy, no alterations in basal ganglia NAA were found (Lu et al., 1996; Keller et al., 2004; Prado et al., 2011). More recently, Mbugua et al. (2016) reported higher absolute NAA levels in HIV+ children initiating ART before 12 weeks compared to a control group comprising mostly (80%) HIV-exposed uninfected (HEU) children.

Findings on glial metabolites in the basal ganglia are less consistent. Keller colleagues and Lu colleagues found lower Cho (Keller et al., 2004) and Cho/Cr (Lu et al., 1996) in HIV+ children than controls. However, other studies found higher basal ganglia Cho/Cr (Ashby et al., 2015) and Ins/Cr (Ashby et al., 2015) in HIV+ children, as well as higher absolute Cho (Mbugua et al., 2016) in HIV+ children who started ART before 12 weeks compared to children who initiated treatment later and a predominantly HEU control group.

Although earlier studies do not report ART use (Pavlakis et al., 1995; Lu et al., 1996), basal ganglia neurometabolite alterations reported subsequently were observed even in HIV+ children on ART (Keller et al., 2004; Gabis et al., 2006; Ashby et al., 2015; Mbugua et al., 2016). However, these studies used relatively small sample sizes (between 8 and 45 HIV+ subjects) spanning a wide age range and starting ART at varying stages of childhood. One study examined basal ganglia neurometabolites in young children who all initiated ART before 18 months of age (Mbugua et al., 2016). In that study, lower CD4/CD8 ratio at enrollment, aged 6–8 weeks, was associated with lower basal ganglia NAA and Cho at age 5 years, despite early ART initiation and viral load suppression.

The aim of this study was to investigate whether immune system impairment in infancy remains associated with lower NAA and Cho in the basal ganglia at age 7 and at age 9 in an expanded group from the same cohort studied by Mbugua et al. (2016). Because increased basal ganglia Cho and NAA in HIV+ children who initiated ART before 12 weeks was attributed to the use of primarily HEU controls (Mbugua et al., 2016), we aimed to compare HIV+ children to HIV-unexposed controls. In addition, although some studies report neurocognitive deficits in HEU children (Van Rie et al., 2008; Kerr et al., 2014), none have investigated the effects of perinatal HIV and ART exposure on neurometabolite levels in childhood. Our second aim therefore, was to investigate differences in basal ganglia neurometabolite levels between HEU and HIV-unexposed children (controls) at ages 7 and 9.

## Materials and Methods

### Participants

Participants included 78 HIV+ children from the Children with HIV Early Antiretroviral therapy (CHER) trial (Violari et al., 2008; Cotton et al., 2013) and 53 HIV-uninfected (HIV-) children from the same community enrolled in a longitudinal neuroimaging study (Holmes et al., 2017) in Cape Town, South Africa.

On the CHER trial, infants with CD4^+^ percentage (CD4%) of at least 25% were randomized to begin ART early (before 12 weeks of age) and have treatment interrupted after either 40 or 96 weeks, or to have ART deferred until CD4% < 20% (25% in the first year) or on presentation of clinical symptoms of disease. A small group with CD4% < 25% were randomized to the early treatment arms only.

The first line ART regimen consisted of Zidovudine (ZDV) + Lamivudine (3TC) + Lopinavir-Ritonavir (LPV/r, Kaletra^®^) (Violari et al., 2008; Cotton et al., 2013). All HIV+ children started ART before 76 weeks of age and received regular clinical and immunological follow-up. All but nine children had plasma HIV RNA below detectable limits (<400 RNA copies/mL) by 2 years of age.

The HIV- children, comprising both unexposed (control) children born to HIV-seronegative mothers (*N* = 32) and HEU children (*N* = 21) born to HIV+ mothers, were recruited from a linked vaccine trial (Madhi et al., 2010). HEU children were exposed to treatment for prevention of mother-to-child transmission (PMTCT), mostly zidovudine antenatally from 28 to 34 weeks and a single dose of nevirapine (NVP) to the mother and zidovudine for a week and a single dose of NVP to the infant.

### Neuroimaging

Neuroimaging was performed without sedation according to protocols approved by the Human Research Ethics Committees of the Universities of Cape Town and Stellenbosch. Parents/guardians provided written informed consent and children provided oral assent. A senior radiologist reviewed all structural scans, and children with abnormalities were excluded from analysis.

At 7 years, children were scanned on a 3T Allegra MRI scanner (Siemens, Erlangen, Germany) using a single channel head coil. At 9 years, a 3T Siemens Skyra MRI scanner with a 32-channel head coil was used.

**FIGURE 1:**
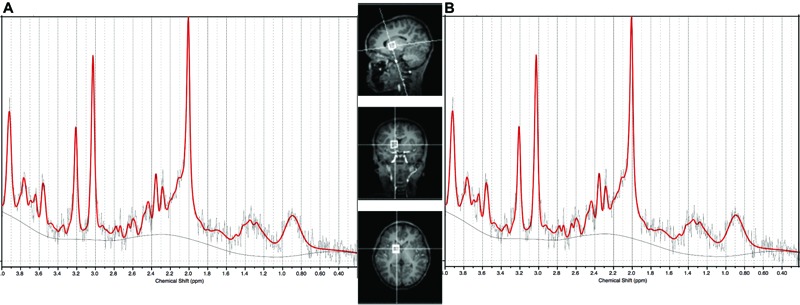
Sample spectrum of median SNR and LCModel output from **(A)** the Allegra scanner used for the 7 year olds (SNR = 8) and **(B)** the Skyra scanner used for the 9 year olds (SNR = 14). Basal ganglia voxel placement shown in center panel.

On both scanners, the protocol included a high-resolution T1-weighted multiecho magnetisation prepared rapid gradient echo acquisition (MEMPRAGE; Van der Kouwe et al., 2008) FOV 224 mm × 224 mm, TR 2530 ms, TI 1160 ms, TE’s = 1.53/3.19/4.86/6.53 ms, bandwidth 650 Hz/px, 144 slices, 1.3 mm × 1.0 mm × 1.0 mm) and single voxel 1H-MRS (PRESS: 1.5 cm × 1.5 cm × 1.5 cm voxel; TR 2000 ms, TE 30 ms, 64 averages) in the basal ganglia with Chemical Shift Selective (CHESS) water suppression. A water reference was acquired in the same voxel without water suppression. On the Allegra, an EPI volumetric navigated (vNav) PRESS (Hess et al., 2011) sequence was used that applies prospective motion- and shim correction throughout the acquisition and has been shown to provide high quality repeatable spectra in young children (Hess et al., 2013). The basal ganglia voxel comprised approximately 60% gray matter and anatomically represents the frontal limb of the internal capsule and part of the caudate nucleus, putamen, and globus pallidus (representative voxel placement shown in **Figure 1**). Shimming was performed over the voxel volume, first automatically using the scanner’s “Advanced” adjustment, then manually if necessary to reduce the spectral linewidths reported by the scanner.

LCModel (Provencher, 2001) was used to perform eddy current correction and to calculate metabolite ratios to creatine, as well as absolute concentrations using the water scaling method (Ernst et al., 1993; Kreis et al., 1993). SPM12 software was used to segment the MEMPRAGE regions corresponding to the SVS voxel into gray matter, white matter, and cerebrospinal fluid (CSF) for partial volume correction and water concentration calculation. Spectra were eliminated if the quality was poor (signal-to-noise ratio < 7, line width at half the peak maximum > 0.07 parts per million as reported by LCModel).

### Statistical Analysis

Because metabolite levels are scanner-dependent and measured in institutional units, data from scans at different ages were treated independently with separate cross-sectional analyses at each age, to examine differences in Cr, Cho, NAA, Glu, and Ins between HIV+/HEU children and HIV-unexposed controls. Linear regression models in the R programming language (R Core Team, 2013) were used, with age at scan, gender, ethnicity, and voxel gray matter content as confounders. To ensure that results were not driven by influential outliers, all analyses were repeated excluding concentration estimates removed more than 1.5 times the interquartile range from the median value for that metabolite.

In the HIV+ group we also used linear regression analysis to examine the relationship of metabolite levels to clinical measures including CD4% at enrollment, CD4/CD8 ratio at enrollment, CD4% closest to scan and incidence of CDC stage C HIV disease.

## Results

At age 7, spectra from 17 HIV+ (38%), 6 HEU (43%), and 7 control children (33%) did not meet the quality control criteria and were excluded. At age 9, data from 1 HIV+ child were excluded. Scans from 37 HIV+ children, 10 HEU children and 13 control children provided data at both 7 and 9 years. **Figure 1** shows the voxel placement and example basal ganglia spectra from the Allegra and Skyra scanners.

Demographic data for subjects included at age 7 and age 9 are presented in **Table 1**. At age 7 there was no difference in birth weight or age between groups. At the 9-year scan there was no difference in birth weight between groups, but the HIV+ children were younger than the control (*p* = 0.0003) and HEU (*p* = 0.00004) children.

**Table 1:** Biographical data for HIV+, HIV-exposed uninfected (HEU), and HIV-unexposed uninfected controls scanned at 7 and 9 years.

Age 7 (*N* = 80)	HIV+ (45)	HEU (14)	Control (21)
Sex	18 M (40%)	7 M (50%)	11 M (52%)
Age (years)	7.20 ± 0.10	7.22 ± 0.10	7.25 ± 0.16
Birth weight (grams)	2970 ± 437	3096 ± 532	3055 ± 382

**Age 9 (*N* = 103)**	**HIV+ (67)**	**HEU (15)**	**Control (21)**

Sex	32 M (48%)	9 M (60%)	10 M (47%)
Age (years)	9.27 ± 0.24	9.73 ± 0.53	9.62 ± 0.54
Birth weight (grams)	3071 ± 408	3159 ± 459	3153 ± 358^a^

*Values are mean ± standard deviation. ^*a*^Data missing for 1 subject.*

Clinical data for the HIV+ children are presented in **Table 2**. Plasma HIV RNA was undetectable (<400 copies/mL) in 91% of the children (all except 4) at the 7-year and 97% (all except 2) at the 9-year scan. At the 7-year scan 3 children had not yet restarted ART after interruption, but by the 9-year scan all children were on ART.

**Table 2:** Clinical data for HIV+ children scanned at 7 and 9 years.

	Age 7 (*N* = 45)	Age 9 (*N* = 67)
**Enrolment data**		
CD4 (cells/mm^3^)	1676 ± 897	1928 ± 838
CD4%	33.0 ± 11.6	35.5 ± 8.7
CD4/CD8	1.3 ± 0.7	1.4 ± 0.7^a^
**Plasma viral load**		
High (>750,000 copies/mL)	26 (58%)	36 (54%)
Low (400–750,000	19 (42%)	31 (46%)
copies/mL) Suppressed (<400 copies/mL)	0	0
**At scan data^a^**		
CD4 (cells/mm^3^)	1194 ± 507	1067 ± 410
CD4%	36.7 ± 5.8	38.2 ± 7.0
**Plasma viral load**		
High (>750,000 copies/mL) Low (400–750,000	0 4 (9%)	0 2 (3%)
copies/mL) Suppressed (<400 copies/mL)	41 (91%)	65 (97%)
**Treatment-related data**		
Age at ART initiation	14.9 ± 13.0	15.1 ± 14.2
(weeks)	range 6.6–64.3	range 5.9–75.7
Children on interrupted ART	24 (53%)	37 (55%)
Age at interruption (weeks)	70.5 ± 27.3	72.1 ± 28.1
Duration of interruption	Median 35.3 (24.8)	Median 34.6 (27.7)
(weeks)	Range 5.7–277.9	Range 6.0–398.0
Cumulative time on ART (weeks)	326.1 ± 64.1	425.9 ± 83.9 weeks
Age at first PVL suppression	Median 47.0 (57.8)	Median 41.4.3 (32.3)
(weeks)	Range 30.7–285.6	Range 29.1–213.3
Incidence of virological breakthrough	28 (62%)	46 (69%)
**CDC classification**		
A	5 (11%)	7 (10%)
B	7 (16%)	10 (15%)
Severe B	9 (20%)	12 (18%)
C	24 (53%)	37 (55%)
HIV encephalopathy cases	8 (13%)	6 (8%)

*Values are number (percentage of group), mean ± standard deviation, or median (interquartile range) and range when specified. Age and duration of interruption data only for children with treatment interruption. CDC classification data missing for 1 subject. ^*a*^Median interval 63 days from 7 year scan; median interval 22 days from 9 year scan.*

**FIGURE 2:**
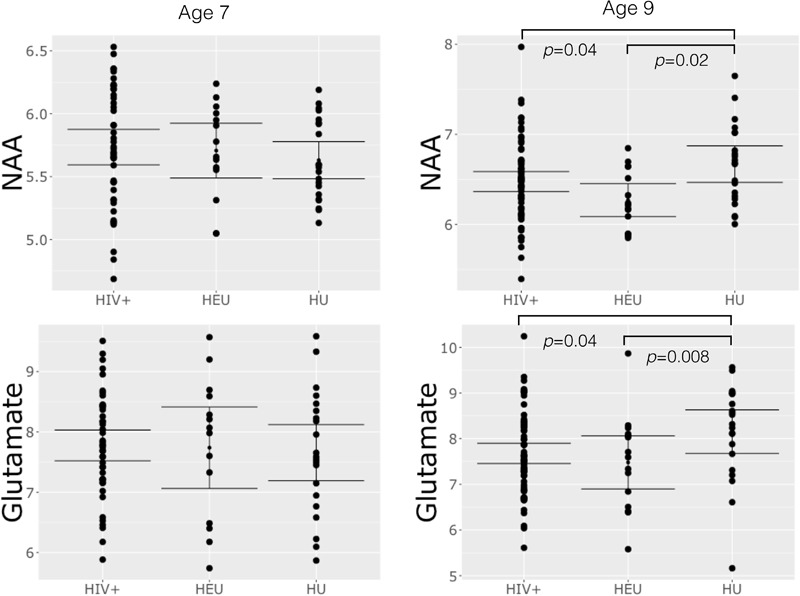
Plots of basal ganglia NAA and glutamate at age 7 and 9 showing mean, 95% confidence intervals and differences in group means significant at *p* < 0.05.

No differences in metabolites relative to the control group were found at age 7 (**Table 3**). However, at 9 years, both HIV+ and HEU had lower NAA and Glu than control children (**Figure 2** and **Table 3**). HEU children also had lower Cr and Cho than control children.

**Table 3:** Unstandardised regression coefficients (B), standard error and *p*-values for basal ganglia absolute metabolite levels relative to control children at age 7 and 9, controlling for sex, age at scan, ethnicity, and voxel gray matter content.

	Age 7 (*N* = 80)						Age 9 (*N* = 103)					
	HIV+ (*N* = 45)			HEU (*N* = 14)			HIV+ (*N* = 67)			HEU (*N* = 15)		
	*B*	*SE*	*p*	*B*	*SE*	*p*	*B*	*SE*	*p*	*B*	*SE*	*p*
**NAA**	0.05	0.11	0.63	0.04	0.15	0.79	**-0.25**	**0.12**	**0.04**	**-0.39**	**0.16**	**0.02**
**Glu**	0.21	0.24	0.39	0.26	0.31	0.41	**-0.46**	**0.21**	**0.04**	**-0.75**	**0.28**	**0.008**
**Cho**	0.03	0.03	0.41	0.07	0.04	0.11	0.03	0.03	0.37	**-0.10**	**0.04**	**0.02**
**Ins**	0.10	0.18	0.59	**-**0.17	0.23	0.46	**-**0.14	0.15	0.35	**-**0.18	0.19	0.35
**Cr**	0.12	0.13	0.40	0.25	0.17	0.10	**-**0.12	0.11	0.30	**-0.42**	**0.15**	**0.005**

*Coefficients significant at *p* < 0.05 marked in bold font.*

**FIGURE 3:**
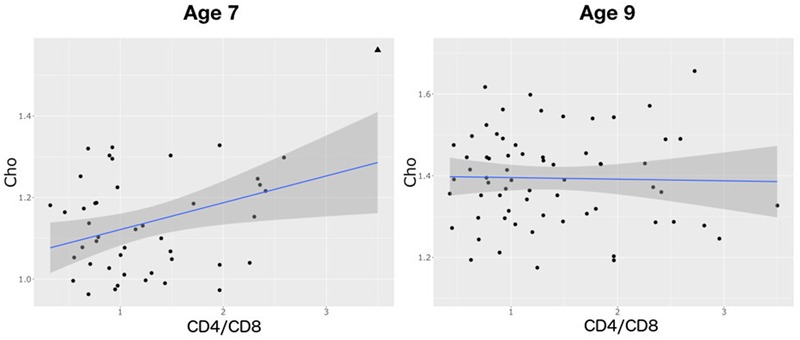
Plots showing a positive relationship between choline and CD4/CD8 ratio at age 7 (*B* = 0.0521, *SE* = 0.02, *p* = 0.01) and none at age 9 (*B* = 0.0026, *SE* = 0.07, *p* = 1.0). Triangle denotes an influential outlier excluded from statistical analysis.

There was no relationship between CD4% at enrollment and neurometabolites at age 7 or 9 years (**Table 4** and Supplementary Table 1). However, at age 7, lower CD4/CD8 ratio at enrollment was associated with lower Cho levels. Also, there was a weak association between lower CD4% at scan and NAA. The same relationships were not evident at age 9, but at this age lower CD4/CD8 at enrollment was associated with lower Ins, and a CDC stage C diagnosis showed a trend-level association with lower NAA and higher Ins. Relationships of CD4/CD8 ratio at enrollment to Cho at ages 7 and 9 are illustrated in **Figure 3**.

**Table 4:** Unstandardised regression coefficients (B), standard error and *p*-values for basal ganglia absolute metabolite levels at age 7 and 9 vs. clinical measures, controlling for sex, age at scan, ethnicity, and voxel gray matter content.

Age 7 (*N* = 45)	NAA			Glu			Cho			Ins			Cr		
	*B*	*SE*	*p*	*B*	*SE*	*p*	*B*	*SE*	*p*	*B*	*SE*	*p*	*B*	*SE*	*p*
CD4% at enrollment	0.0015	0.0065	0.82	**-**0.0183	0.0115	0.12	0.0020	0.0016	0.21	**-**0.0061	0.0079	0.44	0.0026	0.0067	0.70
CD4/CD8 at enrollment	0.0214	0.0809	0.79	0.1124	0.1733	0.52	**0.0521**	**0.0200**	**0.01**	0.0617	0.1289	0.63	0.0141	0.0940	0.88
CD4% at scan	0.0203	0.0113	0.08	**-**0.0161	0.0214	0.46	0.0029	0.0029	0.32	**-**0.0189	0.0138	0.18	0.0068	0.0122	0.58
CDC stage C classification	**-**0.0071	0.1409	0.96	**-**0.3310	0.2533	0.20	0.0073	0.0350	0.84	0.1363	0.1682	0.42	**-**0.0879	0.1463	0.55

**Age 9 (*N* = 67)**	**NAA**			**Glu**			**Cho**			**Ins**			**Cr**		
CD4% at enrollment	0.0005	0.0068	0.9	**-**0.0009	0.0116	0.90	3E-05	1E-03	0.98	0.0121	0.0079	0.13	**-**0.001	0.0065	0.84
CD4/CD8 at enrollment	0.0026	0.0700	1.0	**-**0.0350	0.1270	0.79	0.0026	0.0700	1.00	**0.1613**	**0.0819**	**0.05**	**-**0.1000	0.0670	0.10
CD4% at scan	**-**0.0006	0.0082	0.9	0.0048	0.0139	0.70	**-**0.0018	0.0019	0.35	0.0072	0.0096	0.45	0.0029	0.0078	0.71
CDC stage C classification	**-**0.2082	0.1128	0.07	**-**0.0230	0.1990	0.90	**-**0.0307	0.0275	0.3	0.2370	0.1340	0.08	**-**0.1775	0.1102	0.11

*Coefficients significant at *p* < = 0.05 marked in bold font.*

## Discussion

This study presents SVS basal ganglia neurometabolite data in a larger sample of older HIV+ children than previously studied, 91% of whom had undetectable viral loads at 7 and 97% at 9 years, and all of whom had started ART before 76 weeks of age. Although we find no neurometabolite differences at age 7, lower NAA and Glu is apparent at age 9.

Contrary to findings in a subset of the same children at age 5 (Mbugua et al., 2016), we do not find an effect of immune health in infancy on basal ganglia NAA at either 7 or 9 years, suggesting that subsequent health, or other events during childhood, have a stronger influence on neuronal integrity at these older ages. However, as at age 5, higher CD4/CD8 ratio at enrollment remains associated with higher basal ganglia Cho at age 7, suggesting the effect of early immune health on glial cells may persist for some time.

The *p*-values presented here should, however, be interpreted with caution, as due to the number of statistical tests the family-wise error rate is greater than 5%. Findings significant at *p* < 0.05 would not have survived correction for multiple comparisons. Although the observed associations may therefore represent type I errors, they nevertheless present a starting point for investigation in future studies.

### Decreased NAA and Glutamate in HIV+ Children at Age 9

Similar to previous findings in children without HIVE, at age 7 we find no differences in NAA or other metabolites between HIV+ children and controls. Although NAA/Cr levels have been shown to be decreased in basal ganglia in children with HIVE (Pavlakis et al., 1995; Lu et al., 1996), in HIV+ children without encephalopathy NAA levels have been reported to remain unchanged (Keller et al., 2004). In our own previous study of the same cohort at age 5 years, children who initiated ART before 12 weeks had higher NAA and choline compared to the uninfected group of which 80% were HIV exposed (Mbugua et al., 2016). Notably, at age 9 we find lower NAA and Glu (but not NAA/Cr or Glu/Cr, Supplementary Table 2) in HIV+ children, even though only six children in our 9-year old HIV+ group had a prior HIVE diagnosis.

The reason these differences in NAA and Glu are not observed at age 7 may be that the normal age-related increase in NAA (Holmes et al., 2017) is altered in HIV (Keller et al., 2004), such that NAA is no different from controls at age 7 but has dropped below control levels by age 9. At age 5 (Mbugua et al., 2016), only two of the uninfected children were HIV-unexposed, and the rest HEU, so that we cannot say with any certainty how NAA levels in HIV+ children compared to those of HIV-unexposed children at age 5. This makes it difficult to interpret these findings.

No previous study has reported reduced basal ganglia Glu in HIV+ children; together with reduced NAA this suggests neuronal cell loss. Neurons in the basal ganglia may be more vulnerable than other regions to damage via excitotoxicity because of their greater density of NDMA receptors (Lipton et al., 1991) or because of increased blood–brain barrier damage in these structures associated with high plasma viral load (Avison et al., 2004).

Although in basal ganglia both lower (Lu et al., 1996; Keller et al., 2004) and higher (Ashby et al., 2015) Cho/Cr and Cho have previously been found in HIV+ children, we found no difference in basal ganglia Cho between HIV+ children and controls at either age. In adults, elevated basal ganglia Cho/Cr normalizes after ART (Sailasuta et al., 2012). In these children, ART may have caused normalization of basal ganglia Cho levels, without preventing cell loss. However, the strongest neurometabolite effect observed in regressions with clinical data (B/SE, **Table 4**), is that of Cho at 7 years against CD4/CD8 at enrollment. This replicates the finding in the same cohort at age 5 (Mbugua et al., 2016), suggesting that better immune health in infancy may be associated with a greater cell density in childhood. Although the regression coefficient for Cho is 20 times smaller by 9 years of age, at 9 years the regression coefficient for Ins against baseline CD4/CD8 is similarly large.

An alternative reason for the lack of group differences at age 7 may be the lower SNR obtained from the single channel head coil on the Allegra scanner, resulting in higher standard deviations for metabolite concentrations estimated with LCModel at age 7, even though spectra still met strict quality control criteria. The standard errors for the regressions at each age were comparable, showing similar between-subject variability on each scanner. However, effect sizes were much larger in the 9 year old data (**Table 3**), suggesting greater sensitivity to detect differences on the Skyra. It is notable, however, that no group differences were found at age 7 even when regressions were weighted by the inverse of the metabolite standard deviations, to provide heavier weighting to metabolite concentrations estimated with greater precision by LCModel. Moreover, in our study on the same cohort at age 5 years, we were able to detect in data from the same scanner, with similar spectra of similar quality, and in a smaller sample, metabolite differences between HIV+ children initiating ART before and after 12 weeks (Mbugua et al., 2016).

Alterations in NAA and Glu observed at age 9, but not 5 and 7, suggest that basal ganglia neurons may be particularly affected by perinatal HIV/ART and that neuronal damage may be ongoing in this region despite early ART and viral suppression. The results should be considered exploratory, and suggest that longitudinal investigation should be performed to clarify the timing and persistence of these effects during childhood.

### Reduced Neuronal and Glial Neurometabolites in HEU Children at Age 9

Surprisingly, at age 9 we detect reductions in several neurometabolites in HEU children relative to controls. Notably, regression coefficients for NAA, Glu, and Cr suggest larger reductions in HEU than HIV+ children, and a reduction in Cho is seen relative to controls in HEU but not HIV+ children. Although few neuroimaging studies have been done in HEU children, one diffusion tensor imaging (DTI) study in infants identified a region with high fractional anisotropy in the cerebellum (Tran et al., 2016) and another did not detect DTI or brain volume differences between HEU and control children (Jahanshad et al., 2015). Ours is the first to demonstrate neurometabolite alterations related to HIV/ART exposure. One previous study found elevated white matter Cho/Cr and NAA/Cr ratios in neonates exposed to HIV and ART *in utero*, however, the HIV status of these infants was not determined (Cortey et al., 1994).

Although at age 9 HIV+ children on ART show no difference in Cho level to control children, in HEU children Cho levels are lowered, suggesting that cell density may be reduced. In addition, we found reduced Cr, reflecting lowered energy metabolism, as well as reduced NAA and Glu, possibly reflecting loss of neurons or reduction in normal neurotransmission. Together, these neurometabolite reductions support the suggestion of loss of both neuronal and glial cells.

It is not clear why HIV exposure should be associated with greater basal ganglia neurometabolite reductions than HIV infection. It is likely that in the context of this study HEU children experience some of the same environmental and social stressors as HIV+ children, as well as nutritional and health challenges, but do not have access to the same level of clinical care as the HIV+ children under follow-up on the CHER trial. This may have resulted in poorer neurodevelopmental outcomes for HEU children.

Concern has also been raised about mitochondrial dysfunction in HEU children due to *in utero* and postpartum exposure to ARVs, particularly Zidovudine (Poirier et al., 2003), which may affect neurodevelopment. Neuroimaging in ART-exposed children without symptoms may show abnormalities similar to those in children with congenital mitochondrial disease (Tardieu et al., 2005). Reassuringly, however, the Surveillance Monitoring for ART Toxicities (SMARTT) cohort of the Pediatric HIV/AIDS Cohort Study, including more than 3500 children, recently found no association between *in utero* exposure to ART drugs and cognitive or academic scores in school-age children (Van Dyke et al., 2016).

Although most studies in HEU children under 3 years of age found no neurodevelopmental differences to controls when confounding factors were controlled (Alimenti et al., 2006; Gómez et al., 2009; Williams et al., 2010; Springer et al., 2012; Ngoma et al., 2014), studies from resource-limited settings suggest that HEU infants in Africa demonstrate subtle cognitive and motor impairment, and expressive language delay (Boivin et al., 1995; Le Doare et al., 2012).

Similarly, studies of school-aged HEU children demonstrate subtle deficits compared to control children, particularly in language-related cognitive performance (Van Rie et al., 2008; Kerr et al., 2014; Milligan and Cockcroft, 2017). A recent longitudinal study reported that neurodevelopment of HEU children is initially similar to their HIV-unexposed peers, but neurocognitive performance starts to fall behind that of HIV-unexposed children during childhood (Smith et al., 2017). This might correspond to a lack of normal age-related increase in Cho, Glu, and NAA in the basal ganglia during childhood (Holmes et al., 2017), which could explain our observations of reduced levels relative to control children at age 9.

It is, however, notable that a recent longitudinal study in the same cohort showed no differences in linear metabolite change between HEU and controls between 5 and 10 years, using a smaller sample of 9-year-old children scanned on a different scanner a few months earlier (Holmes et al., 2017). A limitation of the current study is that data were acquired on different scanners at each age, which does not allow straightforward examination of change in metabolites with age. The cross-sectional analyses presented here suggest that the neurometabolite increase with age may not be linear in one or both of these groups, with a difference manifesting only at the later end of this age range in the basal ganglia.

Future work should investigate the association of basal ganglia metabolites with cognitive performance. Although the basal ganglia are critically involved in motor control, they are also implicated in language processing (Booth et al., 2007). It would be interesting to determine whether basal ganglia neurometabolite alterations are related to subtle cognitive and language deficits in HEU children.

## Ethics Statement

This study conforms to the ethical guidelines and principles of the international Declaration of Helsinki, and was approved by the Faculty of Health Sciences Human Research Ethics Committees of both the Universities of Cape Town and Stellenbosch. Parents/guardians provided written informed consent and children oral assent.

## Author Contributions

EM, BL, and AvdK were involved in the study design and acquisition of data. FR, MH, and FL were involved in data and statistical analyses. FR drafted the work and all other authors provided critical revision of the manuscript. FR, MH, EM, BL, ED, and MC provided interpretation of data.

## Conflict of Interest Statement

The authors declare that the research was conducted in the absence of any commercial or financial relationships that could be construed as a potential conflict of interest.
