# Supplementary material for: Perinatal HIV Infection or Exposure Is Associated With Low N-Acetylaspartate and Glutamate in Basal Ganglia at Age 9 but Not 7 Years
Source: Front Hum Neurosci. 2018 May 7;12:145. doi: 10.3389/fnhum.2018.00145 (PMC5949349; doi:10.3389/fnhum.2018.00145)
Supplement: Supplementary file 1 [file Table_1.docx]

Supplementary Table 1: Standardised regression coefficients (β) for basal ganglia absolute metabolite levels at age 7 and 9 vs clinical measures, controlling for sex, age at scan, ethnicity and voxel gray matter content.

| Age 7 (N=45) | NAA | Glu | Cho | Ins | Cr |
| --- | --- | --- | --- | --- | --- |
| CD4% at enrollment | 0.035 | -0.248 | 0.181 | -0.127 | 0.055 |
| CD4/CD8 at enrollment | 0.133 | 0.031 | **0.288** | -0.042 | 0.144 |
| CD4% at scan | 0.250 | -0.109 | 0.131 | -0.197 | 0.072 |
| Age 9 (N=67) | **NAA** | **Glu** | **Cho** | **Ins** | **Cr** |
| CD4% at enrollment | 0.009 | -0.009 | 0.002 | 0.193 | -0.022 |
| CD4/CD8 at enrollment | -0.085 | -0.013 | 0.044 | **0.297** | -0.162 |
| CD4% at scan | -0.009 | 0.036 | -0.112 | 0.092 | 0.041 |

Coefficients significant at *p*<=0.05 marked in bold font.
