# Supplementary material for: Perinatal HIV Infection or Exposure Is Associated With Low N-Acetylaspartate and Glutamate in Basal Ganglia at Age 9 but Not 7 Years
Source: Front Hum Neurosci. 2018 May 7;12:145. doi: 10.3389/fnhum.2018.00145 (PMC5949349; doi:10.3389/fnhum.2018.00145)
Supplement: Supplementary file 2 [file Table_2.DOCX]

Supplementary Table 2: Unstandardised regression coefficients (B), standard error and p-values for basal ganglia metabolite ratios to creatine relative to HU children at age 7 and 9, controlling for sex, age at scan, ethnicity and voxel gray matter content.

|  | Age 7 (N=80) | | | | | | Age 9 (N=103) | | | | | |
| --- | --- | --- | --- | --- | --- | --- | --- | --- | --- | --- | --- | --- |
|  | HIV+ (N=45) | | | HEU (N=14) | | | HIV+ (N=67) | | | HEU (N=15) | | |
|  | B | std err | *p* | B | std err | *p* | B | std err | *p* | B | std err | *p* |
| NAA/Cr | -0.013 | 0.026 | 0.63 | -0.034 | 0.034 | 0.32 | -0.022 | 0.018 | 0.20 | -0.001 | 0.023 | 1.00 |
| Glu/Cr | 0.004 | 0.043 | 0.93 | -0.026 | 0.055 | 0.65 | -0.044 | 0.028 | 0.11 | -0.043 | 0.036 | 0.24 |
| Cho/Cr | 0.000 | 0.006 | 0.90 | 0.001 | 0.008 | 0.90 | 0.007 | 0.005 | 0.15 | -0.003 | 0.006 | 0.61 |
| Ins/Cr | 0.001 | 0.033 | 1.00 | -0.059 | 0.043 | 0.20 | -0.008 | 0.023 | 0.72 | 0.014 | 0.030 | 0.62 |
